# Case Report: Malignancy-mimicking imaging appearances of hepatic parasitic diseases

**DOI:** 10.3389/fonc.2026.1814987

**Published:** 2026-05-07

**Authors:** Chunyuan Luo, Hualin Yan, Yang Pu, Yan Luo

**Affiliations:** West China Hospital, Sichuan University, Chengdu, China

**Keywords:** contrast-enhanced ultrasound (CEUS), differential diagnosis of liver tumors, focal liver lesions, hepatic paragonimiasis, hepatic schistosomiasis

## Abstract

This case report delineates a noteworthy diagnostic challenge at the intersection of hepatic infectious disease and oncology: The imaging features of atypical focal hepatic parasitic disease can overlap with those of hepatic malignancies, leading to a risk of misdiagnosis. We present a 38-year-old female with a left hepatic mass. Initial contrast-enhanced CT findings were suspicious for intrahepatic cholangiocarcinoma (ICC), yet serological tumor markers were within normal limits. Subsequent contrast-enhanced ultrasound (CEUS) revealed paradoxical tunnel-like enhancement, typically associated with hepatic paragonimiasis. However, the patient had a history of residence in a historically endemic region for schistosomiasis. Definitive histopathological analysis revealed necrotizing granulomatous inflammation with eosinophilic infiltration and Charcot-Leyden crystals, no schistosome eggs were identified. Based on positive serology for Schistosoma japonicum IgG antibodies and the patient’s history of residence in an endemic region, a presumptive diagnosis of focal hepatic schistosomiasis was rendered. This case underscores that when imaging and serological findings are discordant, broadening the differential diagnosis to include such atypical parasitic infections is crucial to avoid misdiagnosis and guide appropriate therapy.

## 1 Introduction

The differential diagnosis of focal liver lesions remains one of the core challenges in clinical radiology and oncology practice (1). Certain chronic parasitic infections, such as schistosomiasis, typically present with diffuse hepatic involvement, characterized by imaging features like periportal fibrosis (2). However, when a parasitic infection deviates from its typical course and forms an isolated inflammatory fibromatous granuloma, it may present as a poorly defined solid lesion on contrast-enhanced imaging (3). The hemodynamic and morphological characteristics of such atypical lesions often overlap with imaging features of primary liver tumors and infectious lesions, creating significant diagnostic ambiguity (4).

This case report describes a 38-year-old female patient who presented with a “left hepatic mass detected over one month prior.” contrast-enhanced CT suggested intrahepatic cholangiocarcinoma (ICC). Subsequent tumor marker tests were within normal limits, and contrast-enhanced ultrasound (CEUS) raised suspicion of hepatic paragonimiasis. The final diagnosis was a presumptive one of focal hepatic schistosomiasis.

## 2 Case presentation

A 38-year-old woman with no significant past medical history was referred for evaluation of a left hepatic mass. The lesion was incidentally detected one month earlier during an outpatient workup for cholecystitis and cholelithiasis. The patient is a lifelong resident of Sichuan Province, China, a region historically endemic for Schistosoma japonicum infection, although active transmission has been largely controlled (5). She denied travel to other endemic areas, consumption of undercooked crustaceans, freshwater wading, occupational exposure to stagnant water, exposure to viral hepatitis, or significant alcohol consumption. Since the initial discovery, she has remained entirely asymptomatic, reporting no abdominal pain, fever, jaundice, or unintended weight loss.

Laboratory evaluation on admission was unremarkable. The complete blood count was normal, the percentage of eosinophils was 6.4%(absolute eosinophil count was 0.38 × 10^9^/L), with a reference value of 0.4% -8.0%. Liver function tests (ALT 24 U/L, AST 21 U/L, total bilirubin 9.3 μmol/L, albumin 40.4 g/L) and hepatitis B virus serology were also within normal limits.

**Figure 1:**
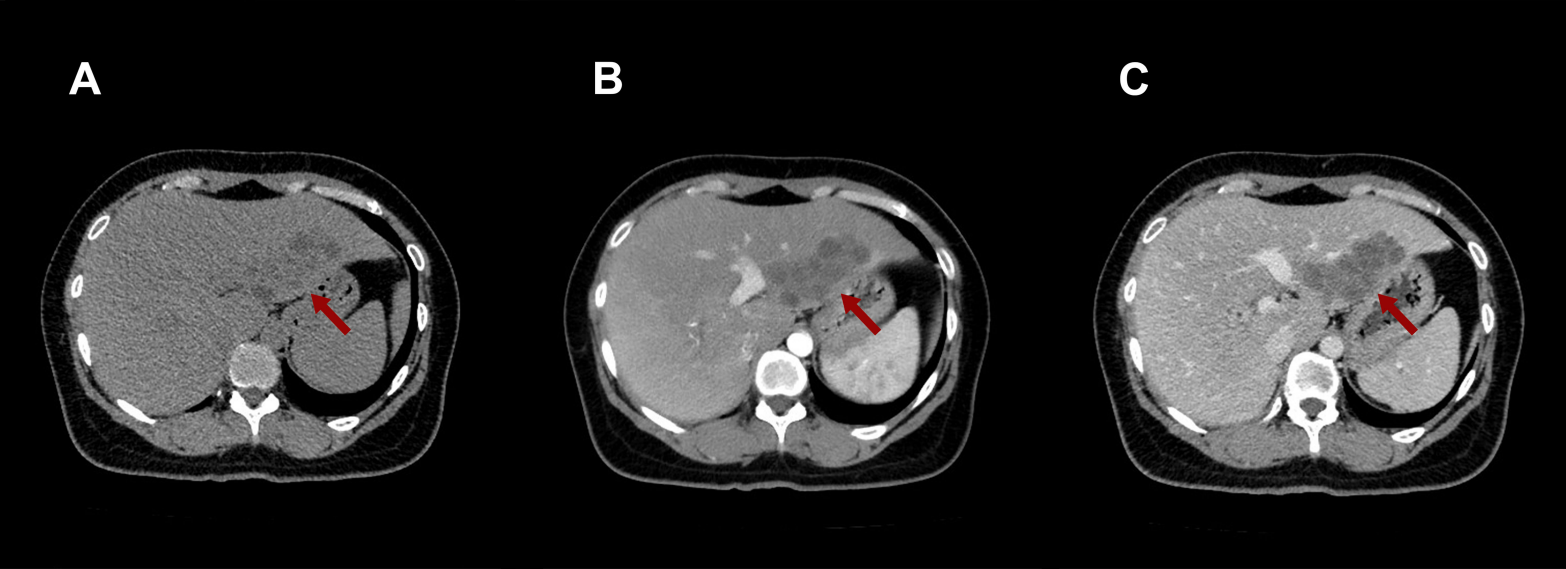
Upper abdominal CT imaging examination. **(A)** Plain scan showed a low-density mass in the left lateral lobe of the liver with blurred boundary. **(B)** The arterial phase showed mild septal-like enhancement. **(C)** Mild enhancement persisted during the venous phase. (The mass is indicated by red arrows in all panels.).

**Figure 2:**
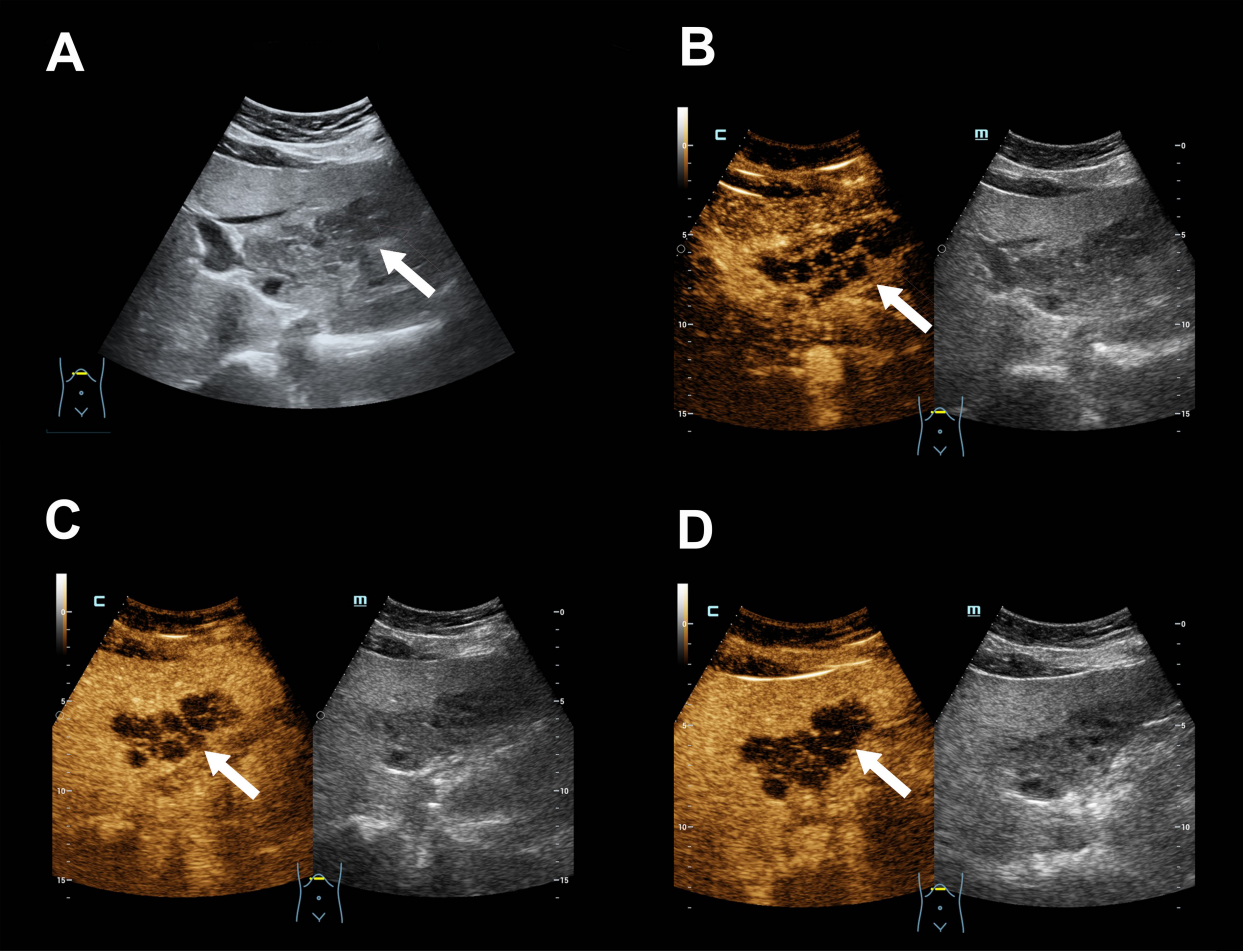
Liver contrast-enhanced ultrasound (CEUS) imaging. **(A)** A hypoechoic mass with indistinct margins, irregular contour, and heterogeneous internal echoes in the left lateral lobe of the liver. **(B)** Arterial phase (12 seconds): sparse punctate-linear enhancement within the mass, appearing isoenhancing. **(C)** Portal phase (69 seconds): the mass shows slight hypoenhancement. **(D)** Late parenchymal phase (136 seconds): the mass remains slightly hypoenhancing, with most of the lesion lacking enhancement and exhibiting a tunnel-like appearance. (The mass is indicated by white arrows in all panels.).

Initial diagnostic imaging with contrast-enhanced CT of the abdomen revealed a 6.8 × 3.6 cm hypodense mass with ill-defined borders in segments II/III of the liver (**Figure 1**), The mass demonstrated mild, progressive, septal-like enhancement from the arterial to the delayed phase, a pattern consistent with fibrous-rich lesions. Radiologists are first alert to this intrahepatic cholangiocarcinoma (ICC). Consequently, a full serum tumor marker panel was analyzed but returned normal results for alpha-fetoprotein (AFP), carcinoembryonic antigen (CEA), carbohydrate antigen 19-9 (CA19-9), and CA-125.For further characterization, contrast-enhanced ultrasound (CEUS) was subsequently performed (**Figure 2**), which revealed an atypical vascular pattern within the lesion: sparse, punctate-to-linear arterial-phase enhancement (at 12 seconds) that washed out in the portal (69 seconds) and late (136 seconds) phases, leaving large non-enhancing areas that collectively generated a pseudo-tunnel-like appearance. This finding redirected the differential diagnosis toward an infectious or inflammatory process, notably hepatic paragonimiasis, thus presenting a clear diagnostic dilemma between neoplastic and infectious etiologies.

Given the persistent diagnostic uncertainty between a potential malignancy and an atypical infection, percutaneous biopsy was initially recommended to clarify the diagnosis. However, the patient expressed a strong preference for direct surgical resection. After detailed consultation and confirmation that there were no contraindications to surgery, the patient’s choice was respected, and she underwent surgical resection, which served the dual purpose of establishing a final diagnosis and achieving complete lesion removal. Intraoperative exploration confirmed a firm, yellowish mass approximately 7 cm in diameter in segments II/III, which was adherent to the left portal pedicle. No other abnormalities were identified in the liver or adjacent structures.

**Figure 3:**
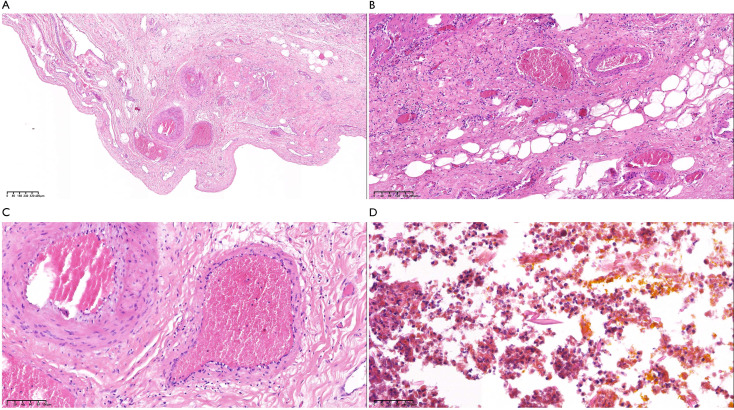
Histopathological examination of the resected specimen (hematoxylin and eosin stain). **(A-C)** (×40, ×100, ×200) Extensive tunnel-like necrosis with granulomatous inflammation and dense eosinophilic infiltration. **(D)** (×400) Charcot-Leyden crystals within the inflammatory exudate. No schistosome eggs were detected.

Histopathological examination revealed extensive necrotizing granulomatous inflammation with eosinophilic infiltration and Charcot-Leyden crystals (**Figure 3**), highly suggestive of a parasitic infection, but no schistosome eggs were definitively identified. To further exclude other endemic parasitic infections in the region, serological tests for echinococcosis (hydatid disease) and clonorchiasis (Clonorchis sinensis) were performed, both of which returned negative results. In the context of the positive schistosomiasis serology, negative results for other regional parasites, the consistent histopathological findings, and the patient’s residence in a historically endemic area, a presumptive diagnosis of focal hepatic schistosomiasis was rendered. Postoperatively, no antiparasitic therapy was administered, as the patient remained asymptomatic and no additional hepatic lesions were identified. At the 5-month postoperative follow-up, the patient’s liver function tests had returned to normal ranges, abdominal ultrasound revealed no new hepatic lesions, and the surgical site showed excellent healing.

## 3 Discussion

Schistosomiasis is a parasitic disease caused by infection with schistosomes, primarily including Schistosoma japonicum, Schistosoma haematobium, and Schistosoma mansoni (6, 7). Its typical pathological and radiological manifestations have been well characterized. Adult parasites reside in the portal venous system, where their eggs are released into the bloodstream and become lodged in small branches of the hepatic portal veins (8, 9). This triggers granulomatous inflammation centered around the eggs, followed by secondary fibrosis. During the chronic phase, ultrasound commonly reveals increased echogenicity and enhanced echoes within the liver parenchyma, presenting a characteristic “map-like” or “lattice-like” appearance (2). At CT, liver manifestations of Schistosoma japonicum infection include linear, reticular, or “tortoiseshell-like” calcifications distributed along portal vein branches, as well as calcification of the liver capsule (10). These characteristic imaging features essentially represent the macroscopic manifestation of extensive, diffuse hepatic fibrosis, typically involving the entire liver.

The present case is among the few reported instances in which a focal chronic hepatic parasitic lesion was initially suspected of being a neoplastic disease (ICC) as well as another parasitic infection (hepatic paragonimiasis). To our knowledge, only a limited number of literature reports have documented focal manifestations of hepatic schistosomiasis. Cesmeli et al. first described focal manifestations of acute hepatic schistosomiasis (11), while Passos et al. first reported focal pancreatic schistosomiasis (12). The misdiagnosis of this case based on imaging findings is not an isolated incident. Ye et al. described focal chronic hepatic schistosomiasis caused by Schistosoma japonicum and documented its misdiagnosis as pancreatic cancer metastasis on PET/CT imaging (13). Wei et al. reported a case of a schistosomiasis granuloma at the hepatic hilum that was misdiagnosed as hilar cholangiocarcinoma (14). The imaging findings and clinical decision-making process in that case were highly similar to those of the present case. Notably, CA19–9 levels were markedly elevated in that patient, whereas all tumor markers remained within normal limits in the present case, reflecting the heterogeneous impact of schistosomiasis-related hepatopathy on tumor marker profiles. Together, these cases highlight that in regions endemic for schistosomiasis, the possibility of a schistosomal granuloma should be considered in the differential diagnosis of radiologically atypical hepatic lesions when malignancy cannot be confidently excluded.

The fundamental challenge in imaging diagnosis of focal liver lesions stems from the phenomenon of “different pathologies sharing similar imaging appearances.” This occurs when disparate disease processes produce lesions with analogous histological matrix and hemodynamic features, leading to overlapping imaging phenotypes. The deep-seated reason for the misdiagnosis in this case is twofold.

On a morphological level (exemplified by CT), both the desmoplastic reaction in ICC and the dense fibrous encapsulation in chronic schistosomal granulomas are predominantly composed of abundant fibrous tissue, which characteristically exhibits slow, persistent contrast retention on enhanced scans, manifesting as “delayed septal enhancement” (15, 16). In this case, the CT finding of progressive septal-like enhancement from the arterial to the delayed phase directly correlated with the histopathological finding of dense fibrous tissue surrounding the granuloma (**Figure 3**). Consequently, these two entities may be indistinguishable by CT alone.

On a microcirculatory perfusion level (exemplified by CEUS), the enhancement pattern is dictated by the internal vascular distribution and necrosis. The schistosomal granuloma, with its ischemic necrotic core surrounded by a hyperemic inflammatory rim, presents on CEUS as sparse peripheral punctate enhancement in the arterial phase, rapid washout in the portal phase, and persistent non-enhancement in the core. This creates a composite image of an anechoic “tunnel” with an enhancing “wall.” This pattern bears a striking resemblance to the enhancement characteristics of true necrotic tracts caused by parasite migration and their surrounding inflammatory walls in hepatic paragonimiasis (17), leading directly to the diagnostic confusion. The “pseudo-tunnel-like” appearance observed in this case—defined as a central non-enhancing region with peripheral punctate enhancement in the arterial phase followed by rapid washout—is not pathognomonic for paragonimiasis alone. Similar patterns can be observed in other necrotizing or fibrotic lesions, including organizing abscesses, tuberculosis, and certain fibrotic tumors.

Several limitations should be acknowledged. First, no schistosome eggs were identified on histopathology, which would have provided definitive confirmation. This may be due to low parasite burden, uneven egg distribution, or prior egg degeneration. Second, since no stool examination for parasites or serological testing for lung fluke infection was performed, the possibility of this disease cannot be completely ruled out. Third, the patient underwent direct surgical resection without preoperative biopsy; thus, the diagnostic utility of percutaneous biopsy for such atypical lesions remains unclear.

## 4 Conclusions

This case demonstrates that an atypical focal hepatic parasitic lesion can present with imaging findings that overlap with those of hepatic malignancies. For a solid liver mass with negative tumor markers and nonspecific imaging features, parasitic infection may be considered as a differential diagnosis, even when a clear epidemiological history is lacking. Histopathological examination remains the definitive diagnostic reference. If non-invasive tests yield inconclusive results, tissue sampling—by percutaneous biopsy or surgical resection—may be considered to reduce the risk of diagnostic error and to inform appropriate clinical decisions.

## Data Availability

The original contributions presented in the study are included in the article/supplementary material. Further inquiries can be directed to the corresponding author.
